# My Fault? Coworker Incivility and Organizational Citizenship Behavior: The Moderating Role of Attribution Orientation on State Guilt

**DOI:** 10.3389/fpsyg.2021.683843

**Published:** 2021-07-01

**Authors:** Qiong Wang, Xiaofei Teng, Zijun Cai, Yi Qu, Jing Qian

**Affiliations:** ^1^School of Labor and Human Resources, Renmin University of China, Beijing, China; ^2^Business School, Beijing Normal University, Beijing, China

**Keywords:** coworker incivility, state guilt, organization citizenship behavior, internal attribution orientation, discrete emotions

## Abstract

The effect of workplace incivility on the behavior of individuals has been a widespread concern in recent years. Previous studies have largely linked uncivilized workplaces to discrete emotions such as anger and frustration, as well as negative behaviors such as withdrawal and aggression. However, few studies have focused on the specific role of introverted discrete emotions (i.e., guilt). At the same time, the role of individual differences (i.e., attribution orientation) has not been paid enough attention. Based on the attribution theory, this study examines how coworker incivility influences the organizational citizenship behavior (OCB) of individuals and the moderating role of internal attribution orientation on this process. Using the data of 109 employees for 10 consecutive working days as samples, we employed the PROCESS macro and MPLUS to examine our hypotheses. The results indicated that coworker incivility experience was positively related to the state guilt of employees only when they were high in internal attribution orientation rather than low. State guilt, in turn, was positively related to their OCB. This study expands the research of emotional response to uncivilized experience and provides a new perspective to understand the relationship between workplace incivility and potential positive outcomes. The implications of the general findings are discussed.

## Introduction

The workplace is no longer considered as a rational and logical space, where abusive supervision, workplace violence, and other negative interpersonal interactions are common phenomena. These negative workplace interactions can pose a significant cost to the organization in the long term. Compared with other forms of interpersonal mistreatment, incivility is considered as a milder form of aggression, with typical characteristics of lacking politeness, rudeness, and disrespect for others, which violates the criterion of mutual respect (Andersson and Pearson, 1999). Because incivility lacks a clear intention to harm (Kabat, 2012), this kind of low-intensity abusive behavior is extremely prevalent in the workplace, causing a more serious impact on employees (Andersson and Pearson, 1999; Lim et al., 2008). For example, customer incivility may result in the emotional exhaustion of employees and may further prolong the negative mood state after the customer service episode (Wang et al., 2011). Using a sample of college students who acted as service providers, Rupp and Spencer (2006) demonstrated that they immediately experienced higher levels of anger following interactions with an impolite customer. Besides, some studies pointed out that workplace incivility overlaps with interactional injustice which also includes the conception of the violation of workplace norms for mutual respect (Koopmann et al., 2015), and the occurrence of a justice-related event gives rise to emotional reactions (Barsky et al., 2011). Just like being denied justice, employees responded with outward-focused emotions, such as anger and hostility, when experienced incivility and generated more subsequent retaliatory behaviors (Barclay et al., 2005).

Focusing on organization insiders, coworker incivility behavior has become one of the main sources of uncivilized experience in the workplace. Previous studies have found that experiencing workplace incivility from colleagues, such as being treated in rude or condescending manners, can damage the targeted emotions of individuals (Cortina et al., 2001). Such discrete emotions have unique appraisal patterns, motivational functions, and behavioral associations and act as motivators for their subsequent behaviors. Specifically, consistent with the cognitive appraisal model (Lazarus, 2001), the emotional elicitation process begins with the primary evaluation of an event. According to the evaluation of the event with well-being and goal attainment, individuals can generate a high intensity of emotional reaction (Dallimore et al., 2007), which in turn activates behavioral motivation and response to the event, resulting in specific emotion-driven behavior. When employees encounter coworker incivility, they will make judgments based on cognition and ethical standards and generate a high intensity of negative emotional response When perceiving the loss of personal resources or inconsistent with their cognition, such emotional response may lead employees to be more inclined to engage in counterproductive work behaviors (CWBs) (Penney and Spector, 2005), while some even change jobs to get away from the incivility (Lim et al., 2008).

Existing studies addressing workplace incivility have focused on the negative effects of emotions and work behaviors of employees. Although Yue et al. (2017) made an interesting discovery, employees with negative emotions caused by customer mistreatment were more likely to engage in helping behaviors toward colleagues and customers the next day when they were high in customer orientation and when cumulative customer mistreatment was low. Scant attention has been given to the experience and repercussions of incivility when feelings evoked are other than negative extroverted emotions (e.g., anger) and the role of individual differences. Guilt, unlike anger, is a self-evaluative, inward-focused emotion (Frijda, 1988). Lewis (1971) described guilt as a negative emotion of self-concern, which is mainly derived from the concern about how his/her own behavior of an individual has a negative impact on others and may lead to certain emotional behaviors. As a personal trait, attribution orientation is an important basis for the generation of individual cognition and emotion and affects following motivation and behaviors (Sullivan and Weiner, 1975). Internal attribution orientation refers to the individuals who have a high degree of self-concern and tend to attribute the cause of things to themselves, which leads to certain emotions and behaviors.

Consistent with the source of guilt, there may be a potential relationship between internal attribution orientation and guilt. Therefore, we would like to explore whether coworker incivility will have different effects on cognition, emotional response, and the subsequent behaviors of individuals. More importantly, with the growing interest in understanding the role of individual differences in shaping behavioral reactions to incivility (Holtz and Harold, 2013), addressing such questions should merit research attention.

Therefore, based on the theory of affective event theory and cognitive theory of emotion, this study views coworker incivility as an affective event, which can stimulate a series of cognition and evaluation of the individual and generate specific discrete emotions (i.e., guilt) according to the results to deal with the occurrence of the event. At the same time, such emotion directly leads to affect-laden behaviors, such as organizational citizenship behavior (OCB). Besides, previous studies have proposed that the process of cognitive evaluation will be affected by individual and environmental characteristics (Cortina and Magley, 2009). Therefore, this study completely considers the role of individual differences and predicts that individual attribution orientation will affect individual event evaluation and emotion generation.

**Figure 1:**
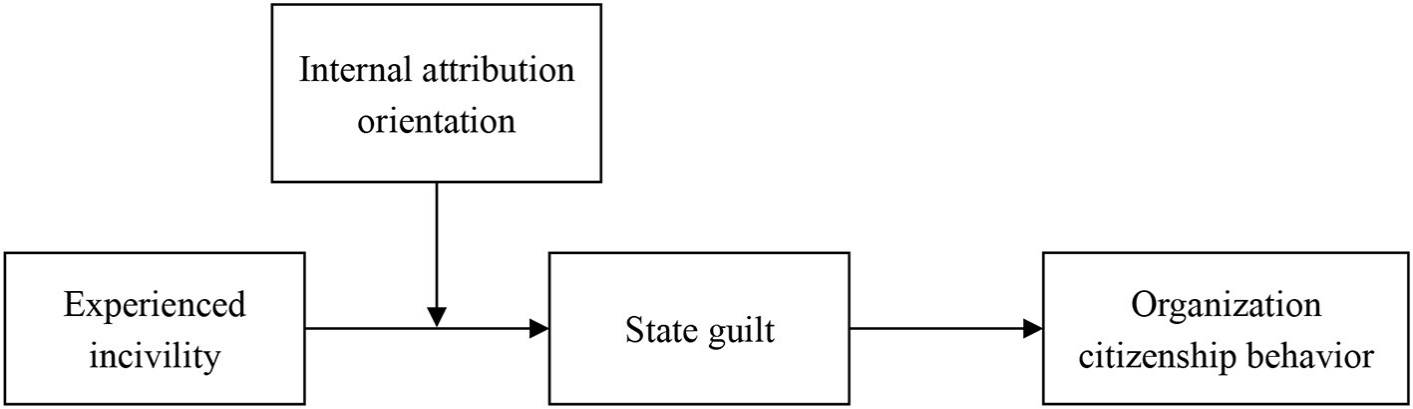
Theoretical model.

Altogether, this study develops a model to indicate the relationships between the coworker incivility and the behaviors of employees and further clarifies the moderating role of guilt and the influence of individual attribution orientation on the strength of these indirect relationships (see Figure 1). By doing so, we contributed to the literature in several ways. First, we jumped out of the limitations of extroverted discrete emotions, such as anger, and explored the role of guilt, an inward-focused emotion, in the relationship between the coworker incivility and the behavior of employees, which enriches the explanation of emotional response for incivility. Second, we paid attention to the role of individual differences and found the important boundary role of individual attribution orientation in the emotional response of incivility, which more comprehensively explains the reasons for the individuals to generate specific emotions and behaviors. Finally, the results of this study indicate that negative events may also bring positive behavioral outcomes, thereby providing a new theoretical direction and perspective for future studies.

## Theory and Hypotheses

### Coworker Incivility and State Guilt: the Moderating Role of Internal Attribution Orientation

Affective event theory posits that positive and negative affective workplace events trigger emotional states that influence the attitudes and behaviors of employees. A previous study found that incivility frequency correlates with anger (Bunk and Magley, 2013). Porath et al. (2007) proposed that people who experience incivility may feel angry because their self-esteem is impaired or their identity is threatened. However, considering the ambiguity of the intention of workplace incivility, some scholars suggested that the emotional response to uncivilized behavior may not be only external negative emotion. As an internal concentrated emotion, guilt may also become a manifestation of a discrete emotional response of incivility.

The generation of individual emotions can be well-explained by the cognitive theory of emotion, which claims that emotions are generated from the cognition and evaluation of stimulus situations or things (Schachter and Singer, 1962), and the cognitive process is the key factor to determine the nature of emotions. Therefore, when faced with stressful events (i.e., incivility), a series of cognitive evaluation results of individuals determine which specific discrete emotions will be used to deal with the event (Lazarus, 2001). Specific evaluation models shape different discrete emotions. When the event is seen as being caused by an entity outside of oneself, they are prone to be more angry and hostile. However, some researchers pointed out that accountability is also an important indicator of cognitive evaluation. Accountability refers to who is responsible for a stressful event. Therefore, guilt occurs when an event is thought to be self-inflicted, and this process depends largely on the characteristics of the individual and the environment (Cortina and Magley, 2009).

Based on these perspectives, we suggested that attribution theory is well-suited to explain the emotional reaction of experienced incivility. Attribution theory is based on the notion that individuals seek to understand the causes of significant events in their lives, particularly when they are important, negative, and/or unexpected (Heider, 1958; Weiner, 1985). This causal reasoning process is related to the generation of specific emotions. Individual attribution orientation will directly affect their cognitive evaluation of uncivilized behavior, resulting in completely different emotional behaviors. Specifically, individuals with internal attribution tend to be accompanied by a high degree of concern for others and reflection on their own responsibility. They are likely to consider whether their own behavior has led to such uncivilized treatment, resulting in self-blame and guilt.

Taken together, we proposed that individual internal attribution affects the relationship between coworker incivility and guilt of employees such that experienced incivility from peers will be positively related to state guilt among those high on internal attribution orientation. Although there is no direct evidence provided for this proposition, studies have shown that personal characteristics are the decisive factor in cognitive assessment and emotional response (Cortina and Magley, 2009), which provides some indirect support for this study. Based on the above findings, we believed that the relationship between coworker incivility and guilt of employees is contingent on the level of internal attribution orientation of an individual. Accordingly, we expected the following:

***Hypothesis 1:****Internal attribution orientation moderates the relationship between coworker incivility and state guilt such that this relationship is positive when the internal attribution orientation of individuals is high as opposed to low*.

### State Guilt and Organizational Citizenship Behavior

Moving on to the consequences of emotional reactions, as part of AET, Weiss and Cropanzano (1996) pointed out that the behavior and performance of employees are related to these reactions. Hareli et al. (2005) had suggested that emotion is a powerful predictor of behavior.

Guilt, unlike anger, is a self-evaluative, inward-focused emotion that arises when one accepts the blame for some misconduct (Frijda, 1988). This self-conscious emotion often stems from the attention to some negative behaviors and can lead to specific emotional behaviors. Previous studies have shown that when individuals experience guilt, they are more likely to lead to thoughts and motivation that are centered on the potential for restoring the situation (Lindsay-Hartz et al., 1995) and motivate them to actively make remedial actions (e.g., admitting mistakes, apologizing, and changing behaviors) (Tangney et al., 2007). Such prosocial behaviors are the remedial result of the negative behaviors they experienced, which helps to reduce the guilt-induced distress of individuals (Miron et al., 2006; Lickel et al., 2011). Similarly, in the workplace, negative experiences can also stimulate the reflection of employees on their responsibilities, which may lead to guilt and self-blame. Accordingly, with attempts of restitution and making amends, they will make more OCBs to prevent the recurrence of similar events. Hence, we proposed that:

***Hypothesis 2:****State guilt is positively related to OCB*.

### An Integrative Moderated Mediation Model

Taken together, the above considerations sketch a complex picture of the relationship between experienced incivility and behaviors of employees which suggests that state guilt will mediate the associations between experienced incivility and behaviors of employees and that the strength of such indirect relationship hinges on the level of internal attribution orientation of individuals. Specifically, when employees experienced incivility from peers, those with high internal attribution orientation tend to reflect on their own responsibility and think more about whether their own behavior has led to such uncivilized treatment, resulting in self-blame and guilt, and will find ways to make up for it by making beneficial actions, such as OCB.

To examine this mechanism as a whole, we, therefore, specified a first-stage moderated mediation model (Preacher et al., 2007), which suggests that internal attribution orientation will moderate the mediation effect of guilt emotions on the association between experienced incivility and behaviors of employees. This model integrates a relational mediator (i.e., guilt), a relational moderator (i.e., internal attribution orientation), and a relational outcome (i.e., OCB) into an overarching framework and well represents our proposed perspective of attribution orientation. Therefore, we hypothesized the following:

***Hypothesis 3:****The indirect effects of experienced incivility on OCB via state guilt are moderated by internal attribution orientation such that these indirect associations are positive when internal attribution orientation is high*.

## Method

### Sample and Procedures

With the help of MBA students, we randomly selected 120 full-time employees from various industries in China as candidates. We first introduced the purpose and procedures of this study and sent an online questionnaire to participants to collect demographic information and measure their negative affective and attribution orientation. Participants were then asked to report their daily experience for 10 consecutive workdays. The daily online survey was sent twice a day and was used to measure experienced incivility, state guilt, and OCB. Participants completed the survey questionnaire voluntarily, and their responses were matched with identification numbers. Respondents were assured that their responses were confidential. All data were collected before the coronavirus disease 2019.

The final sample consisted of 120 participants and 109 participants who completed all 10 questionnaires, which led to 1,054 data points at the within-individual level. Given the maximum of 1,200 possible data points (i.e., 120 participants × 10 days), the overall response rate was 87.8%. Of the 109 participants, 41.7% participants were males (SD = 0.50); the average length of working was 4.32 years (SD = 3.29).

### Measures

The scale items we used in this study were originally written in English, and most of them were validated in the Chinese context. Considering that the respondents were Chinese, we followed and applied the standard translation and back-translation procedure suggested by Brislin (1970) in order to obtain Chinese versions of the survey instructions and questionnaires. All ratings were made on a seven-point Likert-type scale ranging from 1 (strongly disagree) to 7 (strongly agree). Table 1 presents the means, SDs, reliabilities, and correlations for the variables studied.

**Table 1:** Means, SDs, reliabilities, and correlations among study variables.

	**Variables**	**Means**	**SD**	**1**	**2**	**3**	**4**	**5**	**6**	**7**	**8**
1	Gender	1.59	0.50	—							
2	Tenure	4.32	3.29	0.11^**^	—						
3	Negative affectivity	4.18	1.38	0.06	0.06	—					
4	Hostility state	2.90	1.66	−0.01	0.04	0.20^**^	—				
5	Experienced incivility	1.95	1.06	−0.29^**^	−0.15^**^	−0.02	0.41	(0.94)			
6	Guilt state	2.67	1.51	−0.06^*^	0.02	0.18^**^	0.71^**^	0.45^**^	(0.91)		
7	Internal attribution orientation	3.94	1.51	−0.11^**^	0.08^*^	0.22^**^	0.00	−0.01	0.05	(0.85)	
8	Organizational citizenship behavior	5.18	1.24	0.13^**^	0.17^**^	0.01	−0.07^*^	0.20^**^	−0.02	0.01	(0.90)

*N range from 105 to 109; gender: male = 1, female = 2; Cronbach's α reliabilities are in parentheses on the diagonal*;

* *p < 0.05*,

** *p < 0.01*,

*** *p < 0.001*.

#### Experienced Incivility

We measured experienced incivility from peers with the seven items based on the work of Cortina et al. (2002). A sample item is “Today, have you been in a situation where any of your co-workers put you down or was condescending to you?” Cronbach's α for this measure was 0.94.

#### State Guilt

Following the procedure of previous empirical researchers, we measured state guilt with a five-item scale validated by Sheikh and Janoff-Bulman (2009). Sample items are “I cannot stop thinking about something bad I have done” and “I feel like apologizing, confessing.” In this study, Cronbach's α was 0.91.

#### Attribution Orientation

Attribution orientation was assessed using a four-item scale developed by Eberly et al. (2017), including internal and external orientation. A sample item of internal orientation is “My work experiences generally reflect an aspect of me,” and Cronbach's α was 0.85. Sample item of external orientation is “My work experiences generally reflect an aspect of my work environment.” In this study, Cronbach's α was 0.82.

#### Organizational Citizenship Behavior

Using a six-item scale validated by Coyle-Shapiro (2002), we assessed OCB of employees. Sample items are “I go out of the way to help colleagues with job-related problems” and “I readily assist supervisor with his/her work.” Cronbach's α for this measure was 0.90.

#### Control Variables

We controlled variables that may potentially influence the perceptions of individuals of experienced incivility. In line with the previous studies (Kabat, 2012), the gender of participants and tenure were controlled. We also controlled the effects of negative affectivity, which was assessed using a four-item scale developed by Watson et al. (1988). Negative affective is a stable and common individual difference characterized by a tendency to experience an emotional state. In fact, studies have shown that trait NA can have a significant impact on the emotional and psychological responses of individuals to work stressors (Berry et al., 2007). Therefore, we controlled this to get more accurate results. In addition, hostility is the most common emotional response to workplace negative behaviors. In order to explore the potential mechanism of introverted discrete emotion on the behaviors of employees, we also controlled the hostility state of participants. We measured hostility by averaging the responses to the six items from the PANAS-X hostility scale (Watson and Clark, 1994). The participants were asked to rate the extent to which they had experienced feelings such as “irritable,” “hostile,” “disgusted,” “scornful,” “angry,” and “loathing” when they suffered from interpersonal incivility. According to the actual experience and feeling, respondents were asked to indicate the extent to their approval of the given emotional description. Response options range from 1 (strongly disagree) to 7 (strongly agree).

#### Analysis Strategy

Given that all variables were self-reported by participants, we first conducted a confirmatory factor analysis (CFA) using the AMOS 23 and Harman's single-factor test to examine the common method bias (CMB) (Podsakoff et al., 2003). Next, we used the MPLUS 8 to examine the mediating role of state guilt in the relationship between coworker incivility and OCB as well as the moderating effects of internal attribution orientation.

### Hypothesis Testing

Means, SDs, and correlations are presented in Table 1. We performed a series of confirmatory factor analyses to establish the discriminant validity of our measurement model. The estimated result of the CFA model is shown in Table 2. The fit statistics of the hypothesized four-factor model indicated acceptable fit: χ^2^ = 975.95; *df* = 164; CFI = 0.95; NFI = 0.94; RMSEA = 0.07. This four-factor model was significantly better than a three-factor model in which internal attribution orientation and state guilt were combined into one factor (χ^2^ = 1773.80; *df* = 167; CFI = 0.90; NFI = 0.89; RMSEA = 0.20), and a two-factor model in which coworker incivility, guilt, and internal attribution orientation were combined into one factor (χ^2^ = 4770.70; *df* = 169; CFI = 0.73; NFI = 0.69; RMSEA = 0.17), providing evidence of the distinctiveness for the constructs. In addition, the single-factor model (χ^2^ = 8490.93; *df* = 170; CFI = 0.50; NFI = 0.44; RMSEA = 0.22) in CFA did not fit the data as well as the baseline model, indicating that the CMB was not serious (Huang, 2012). Following Podsakoff et al. (2003), we further conducted Harman's one-factor test to examine the CMB. The results showed that no single factor emerged, and the first factor did not explain the majority of the variance. Based on the CFA and Harman's one-factor test, CMB is at an acceptable level in this study and has little impact on the study results.

**Table 2:** Results of confirmatory factor analyses.

**Model**	**χ2**	***df***	**CFI**	**NFI**	**RMSEA**
Four-factor model (proposed model)	975.95	164	0.95	0.94	0.07
Three-factor model: guilt state and internal attribution orientation were combined into one factor	1773.80	167	0.90	0.89	0.10
Two-factor model: experienced incivility, guilt state and internal attribution orientation were combined into one factor	4770.70	169	0.72	0.69	0.17
One-factor mode: experienced incivility, guilt state, OCB and internal attribution orientation were combined into one factor	8490.93	170	0.50	0.44	0.22

**Figure 2:**
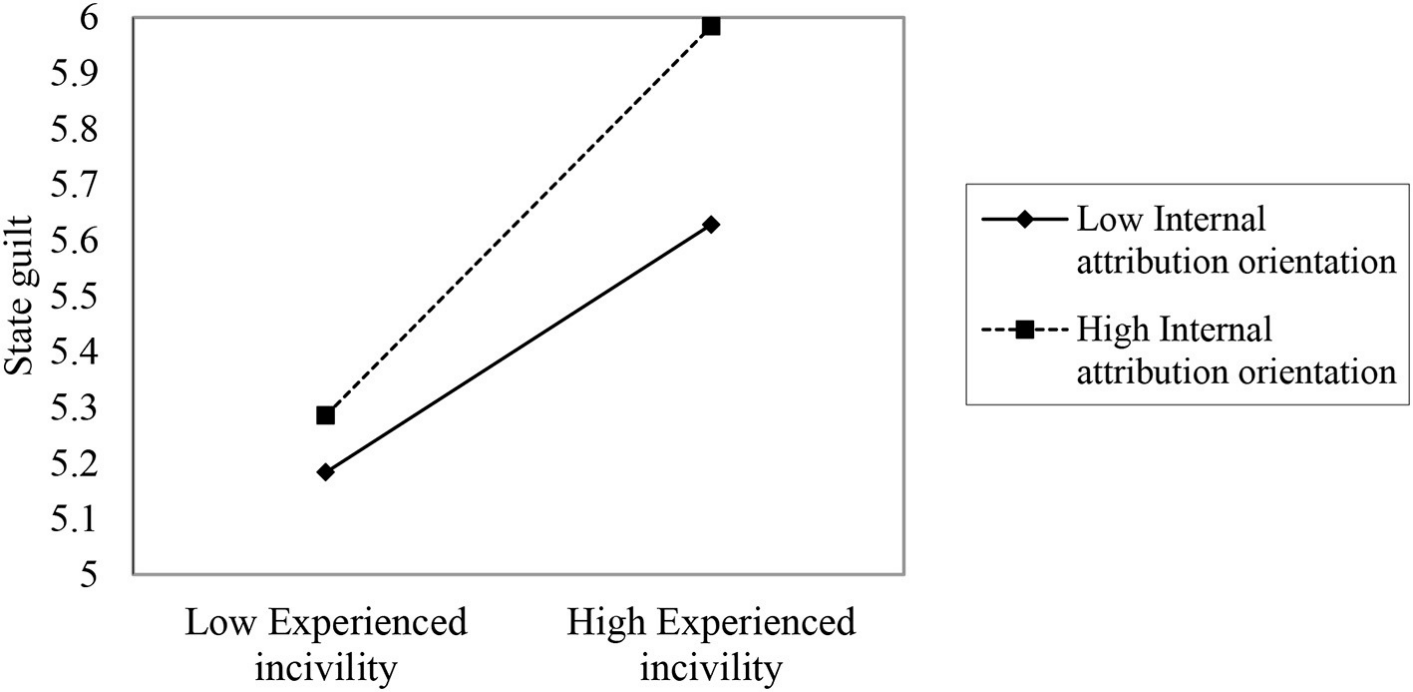
The moderating role of internal attribution orientation.

Hypothesis 1 predicts that internal attribution orientation moderates the relationship between experienced coworker incivility and guilt of employees. As shown in Table 3, the cross-product of experienced incivility and internal attribution orientation was positively associated with state guilt (*b* = 0.11, *p* < 0.05) after considering the control variables and main effects. Following Cohen and Cohen (1985), we plotted the interaction effects and conducted a simple slope analysis at conditional values of the moderators (1 SD above and below the mean). As shown in Figure 2, the simple slope of the relationship between experienced incivility and state guilt was significant and positive under the condition of high internal attribution orientation (*b* = 0.81, *p* < 0.05). Hence, Hypothesis 1 was supported.

**Table 3:** Results for moderation and moderated mediation hypotheses.

**Predictor**	**State guilt**	**Organizational citizenship behavior**
Gender	0.19(0.09)^**^	0.18(0.08)^**^
Tenure	0.03(0.01)^**^	0.05(0.01)^**^
Negative affectivity	0.19(0.03)^**^	−0.01(0.03)
Hostility state	0.56(0.02)^**^	−0.06(0.03)^**^
Experienced incivility	0.70(0.04)	−0.21(0.04)^**^
Internal attribution orientation	0.02(0.03)	0.01(0.06)
Experienced incivility × Internal attribution orientation	0.11(0.05)^**^	
State guilt		0.05(0.29)^**^
*R* ^2^	0.50	0.26
Conditional indirect relationships between experienced incivility and organizational citizenship behavior Moderator value (Effect [95% CI])		
High internal attribution orientation (+1 *SD*)		0.06 [0.00,0.15]
Low internal attribution orientation (−1 *SD*)		−0.06 [−0.14,0.00]

*N range from 105 to 109; gender: male = 1, female = 2*;

* *p < 0.05*,

** *p < 0.01*,

*** *p < 0.001*.

Hypothesis 2 predicts that the guilt of employees is positively related to OCB. As shown in Table 2, after taking the effects of the control variable and the predictor (i.e., coworker incivility) into account, we found that the associations of state guilt with OCB (*b* = 0.05, *p* < 0.05) were positive and significant. Therefore, Hypothesis 2 received support.

Hypothesis 3 predicts the internal attribution orientation to moderate the indirect effects of coworker incivility on OCB as transmitted by state guilt of employees. As shown in Table 3, the results based on the Monte Carlo method showed that the indirect relationship between incivility and OCB was positive and significant (effect = 0.06, 95% CI = [0.00, 0.15]) when internal attribution orientation was high and not significant (effect = −0.06, 95% CI = [−0.14, 0.00]) when internal attribution orientation was low, supporting Hypothesis 3.

The results of this study provide the support for our hypotheses that experienced incivility influences various OCBs of employees through their sense of guilt, and internal attribution orientation determines the direction of such influence.

## Discussion

### Theoretical Contributions

This study makes theoretical contributions in three ways. First, based on the affective event theory and cognitive theory of emotion, we further explored the specific mechanism between coworker incivility and the behavior of employees. Regarding an emotional event, coworker incivility can stimulate a series of cognition and evaluation of the individual and generate specific discrete emotions and emotional-driven behaviors. Despite the fact that most of the previous studies have shown that the experience of incivility can negatively affect the behavior of employees through experiencing strong and intense emotional states, such as hostility (Tice et al., 2004), less information is known about the role of introverted discrete emotion (i.e., guilt) working in workplace incivility. This study finds that guilt may also become one of the emotional reactions of incivility, which will lead to further prosocial behaviors related to compensation (i.e., OCB).

Second, we focused on the role of interpersonal differences in this process. The individual cognitive process is greatly influenced by individual characteristics, and the causal reasoning process is related to the generation of a specific emotion (Lazarus, 2001). Therefore, we suggested that internal attribution orientation is an important boundary condition between coworker incivility and guilt of employees. For individuals with higher internal attribution orientation, they pay more attention to the feelings of others and their own responsibility for unpleasant experiences. Therefore, when facing incivility from peers, they are more likely to be guilt and self-blame, which motivates them to make more OCB for restitution and making amends.

Finally, we enriched the studies on the outcomes of workplace incivility. Previous studies have mainly focused on the negative emotional and behavioral responses of incivility, such as CWB (Sakurai and Jex, 2012), withdrawal behavior (Hanisch and Hulin, 1991), and retaliation behavior (Spencer and Rupp, 2009). However, this may not be the case. Our results show that for those who attribute the unpleasant experience to his/her own responsibility, the feeling of guilt will make them show more positive behavior to make amends and reduce their sense of guilt. This provides a new theoretical direction for future study.

### Practical Implications

Our findings also have several practical implications. For organizations and managers, they should make efforts to construct a harmonious atmosphere in the workplace to reduce the uncivilized behavior. Although the results of this study indicate that guilt may act as an incentive to encourage employees to engage in positive work behaviors with the attempts of making amends. This process has strict boundary conditions. For most employees, the result of workplace incivility is negative, whether silence, exit, or counterproductive behavior. Therefore, it is necessary to establish an inclusive and civilized organizational and interpersonal atmosphere. Organizations can regularly carry out formal or informal team-building activities, which could promote mutual understanding and respect among employees and build good interpersonal relationships. Besides, when workplace incivility occurs, effective managerial interventions are recommended for managers to uncivilized employees. For example, managers can clarify the causes and responsibilities in time through respective conversations and make correct and positive emotional and behavioral guidance.

The employees should improve their judgment and coping ability. When they suffer rude treatment from colleagues, they should adopt appropriate coping strategies and adjust their negative emotions in time and make efforts to repair poor social relationships, such as seeking support from leaders or other colleagues. In addition, individuals can actively participate in relevant training courses organized by organizations or other institutions to systematically learn how to know themselves and how to make correct cognition and judgment on negative events, so as to reduce and regulate their negative emotions and behaviors.

### Limitations and Future Study

Our findings also have several practical implications. First, all constructs in our model were self-rated. The use of self-reports potentially raises concerns about CMB (Podsakoff et al., 2003). However, we attempted to minimize the common method variance by controlling the trait negative affectivity and by stressing to the participants about the anonymity of their responses. Besides, the self-report approach is appropriate in this study. First, the perception of experienced incivility, state guilt, and individual attribution orientation are closely related to individual psychological experience and characteristics, and thus it is appropriate for them to be reported by individuals themselves. Second, the meta-analysis of Berry et al. (2011) also showed that self-report of behavior structure is even better than other ratings, which indicates that it is feasible to capture OCB through the self-reported data. However, future studies could adopt multiple research methods (e.g., behavioral experiments and questionnaire surveys), strengthening the control of employee behavior measurement methods and further exploring the possible behavioral consequences of workplace incivility.

Second, this study did not control the number of social interactions between respondents and their colleagues. The frequency of social interaction and the nature of work may affect the relationship between the experienced coworker incivility and the work behavior of employees because coworker incivility is unlikely to occur when the employees seldom or never see their colleagues. Future studies should control the nature of work or interaction needs of participants to exclude the potential impact on experimental results. Besides, we only paid attention to the influence of coworker uncivilized behavior on the emotion and behavior of employees. However, individuals may receive incivility from multiple sources (e.g., supervisor and customers) in the workplace. Future studies should further explore the effects derived from such different sources.

Finally, this study only focuses on the influence of attribution orientation on the emotions and behaviors of uncivilized employees. However, individual cognition and behaviors may be affected by multiple factors (e.g., personal traits, cultural identity, and environment) (Yang and Diefendorff, 2009; Shao and Skarlicki, 2014). Future study can further investigate the moderating effects of other individual differences and external factors. Besides, previous studies have pointed out that negative interactions in the workplace do not occur within a vacuum and can be mostly witnessed by others (Glomb, 2002; Jóhannsdóttir and Ólafsson, 2004). Therefore, as individuals in the same ecosystem with the perpetrator and the victim, the reactions of bystanders may have a potential impact on attitudes and behaviors of employees, which should be paid more attention in future studies.

## Conclusion

As a result, considering individual differences, this study provides novel insights. The results from this study indicate that internal attribution orientation, as an indispensable boundary, will moderate the mediation effect of guilt emotions on the association between the experienced incivility and the behaviors of employees. This study expands the research of emotional response to uncivilized experience and provides a new perspective to understand the relationship between workplace incivility and outcomes.

## Data Availability Statement

The original contributions presented in the study are included in the article/supplementary material, further inquiries can be directed to the corresponding author/s.

## Ethics Statement

All procedures performed in studies involving human participants were in accordance with the ethical standards of the institutional and/or national research committee and with the 1964 Helsinki declaration and its later amendments or comparable ethical standards with written informed consent from all subjects. This research was approved by the Research Committee at the Business School, Beijing Normal University.

## Author Contributions

QW and XT contributed equally to the development of this article. QW, XT, YQ, and JQ contributed to conception and design of the study. JQ organized the database. XT performed the statistical analysis and contributed most the first draft of the manuscript. ZC, YQ, and JQ wrote sections of the manuscript. QW was responsible for the revision of the manuscript and led the R&R process. ZC gave critical help for the revision. XT, YQ, and JQ also contributed to manuscript revision, read, and approved the submitted version.

## Conflict of Interest

The authors declare that the research was conducted in the absence of any commercial or financial relationships that could be construed as a potential conflict of interest.
