# Corrigendum: My Fault? Coworker Incivility and Organizational Citizenship Behavior: The Moderating Role of Attribution Orientation on State Guilt

**DOI:** 10.3389/fpsyg.2021.741067

**Published:** 2021-08-30

**Authors:** Qiong Wang, Xiaofei Teng, Zijun Cai, Yi Qu, Jing Qian

**Affiliations:** ^1^School of Labor and Human Resources, Renmin University of China, Beijing, China; ^2^Business School, Beijing Normal University, Beijing, China

**Keywords:** coworker incivility, state guilt, organization citizenship behavior, internal attribution orientation, discrete emotions

**Qiong Wang and Zijun Cai** were not included as an author in the published article. The corrected Author Contributions Statement appears below.

QW and XT contributed equally to the development of this article. QW, XT, YQ, and JQ contributed to conception and design of the study. JQ organized the database. XT performed the statistical analysis and contributed most the first draft of the manuscript. ZC, YQ, and JQ wrote sections of the manuscript. QW was responsible for the revision of the manuscript and led the R&R process. ZC gave critical help for the revision. XT, YQ, and JQ also contributed to manuscript revision, read, and approved the submitted version.

In the original article, we neglected to include the funder **Fundamental Research Funds for the Central Universities**, **[2019NTSS09] to ZC**.

The authors apologize for this error and state that this does not change the scientific conclusions of the article in any way. The original article has been updated.

## Publisher's Note

All claims expressed in this article are solely those of the authors and do not necessarily represent those of their affiliated organizations, or those of the publisher, the editors and the reviewers. Any product that may be evaluated in this article, or claim that may be made by its manufacturer, is not guaranteed or endorsed by the publisher.

